# In situ scanning tunneling microscopy study of Ca-modified rutile TiO_2_(110) in bulk water

**DOI:** 10.3762/bjnano.6.44

**Published:** 2015-02-12

**Authors:** Giulia Serrano, Beatrice Bonanni, Tomasz Kosmala, Marco Di Giovannantonio, Ulrike Diebold, Klaus Wandelt, Claudio Goletti

**Affiliations:** 1 Department of Physics, Università degli Studi di Roma “Tor Vergata”, Via della Ricerca Scientifica 1, 00133 Rome, Italy; 2 Institut für Physikalische und Theoretische Chemie, Wegelerstraße 12, 53115 Bonn, Germany; 3 Istituto di Struttura della Materia (ISM), Consiglio Nazionale delle Ricerche (CNR), Via Fosso del Cavaliere 100, 00133 Rome, Italy; 4 Institute of Applied Physics, Vienna University of Technology, Wiedner Hauptstraße 8–10/134, 1040 Vienna, Austria

**Keywords:** alkali earth metals, scanning probe microscopy, solid/liquid interface, titanium dioxide reconstruction

## Abstract

Despite the rising technological interest in the use of calcium-modified TiO_2_ surfaces in biomedical implants, the Ca/TiO_2_ interface has not been studied in an aqueous environment. This investigation is the first report on the use of in situ scanning tunneling microscopy (STM) to study calcium-modified rutile TiO_2_(110) surfaces immersed in high purity water. The TiO_2_ surface was prepared under ultrahigh vacuum (UHV) with repeated sputtering/annealing cycles. Low energy electron diffraction (LEED) analysis shows a pattern typical for the surface segregation of calcium, which is present as an impurity on the TiO_2_ bulk. In situ STM images of the surface in bulk water exhibit one-dimensional rows of segregated calcium regularly aligned with the [001] crystal direction. The in situ-characterized morphology and structure of this Ca-modified TiO_2_ surface are discussed and compared with UHV-STM results from the literature. Prolonged immersion (two days) in the liquid leads to degradation of the overlayer, resulting in a disordered surface. X-ray photoelectron spectroscopy, performed after immersion in water, confirms the presence of calcium.

## Introduction

Metal oxide surfaces (in particular titanium dioxide (TiO_2_) surfaces) covered by an alkaline-earth-metal overlayer have been investigated in recent years in experiments [1–5] and theoretical studies [6], considering applications ranging from nanotechnology [7] to gas sensing [8], as well as catalysis [9] and biomedicine [10–11]. In particular, the high corrosion resistance of titanium to biological environments has stimulated the study of this metal and its oxide for in vivo or in vitro calcium phosphate ceramic growth [12–15]. Recently, the deposition of a thin calcium layer onto TiO_2_ substrates resulted in a prototypical model of the interface responsible for the bone growth by apposition in medical implants [16].

The experiments reported in the literature mostly concern Ca overlayers on a TiO_2_(110) rutile surface prepared under ultrahigh vacuum (UHV) conditions, which is considered to be a model system [10–11]. Ordered Ca layers have been obtained by thermally activated segregation from the bulk [1–5], where calcium was a common bulk impurity in the TiO_2_ samples [10]. A *c*(6 × 2) structure has been proposed for the resulting Ca overlayer based on low energy electron diffraction (LEED) and scanning tunneling microscopy (STM) measurements [1]. Segregation has been reported to produce an additional, differently ordered Ca layer, namely a *p*(3 × 1) structure [2–4]. More controlled and homogeneous Ca overlayers have been grown on a TiO_2_ surface by metal vapor deposition (MVD) technique [5], showing that both MVD and segregation methods can produce a *c*(6 × 2) Ca structure. Finally, theoretical calculations have provided insight into possible preferential adsorption sites for Ca atoms on this surface [6].

The study of the Ca/TiO_2_ interface in ideal (UHV) conditions lacks information about the electronic and structural modifications occurring under more realistic conditions, in particular, in an aqueous environment. Such a study would be an essential step towards a more thorough understanding of the system, leading to the optimization of many related applications. As an example, it was recently demonstrated that a low surface calcium coverage may increase the wetting energy and therefore the surface hydrophilicity of TiO_2_ surfaces [17]. This has very interesting implications for the application of Ti-based biomaterials, since the augmented wettability would enhance the interaction between the implant surface and the biological environment.

In this paper we present an in situ STM investigation of a Ca overlayer thermally grown in UHV on the TiO_2_(110) rutile surface and then immersed in water. The *c*(6 × 2) LEED pattern observed after UHV preparation and the subsequent X-ray photoelectron spectroscopy (XPS) measurements confirm that high-temperature annealing produces a Ca layer by thermal segregation from the bulk. After immersion in high purity water, STM images show quasi one-dimensional rows of bright protrusions aligned with the [001] direction of the TiO_2_(110) surface. The stability of this overlayer in water is discussed.

## Results and Discussion

**Figure 1:**
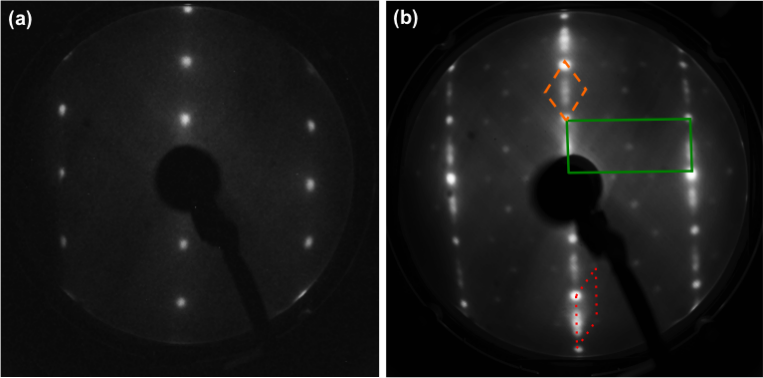
LEED pattern of (a) the clean TiO_2_(110) surface showing the 1 × 1 termination (electron beam energy *E* = 56 eV); (b) the TiO_2_(110) surface after Ca segregation from the bulk, after annealing in UHV at a higher temperature (electron beam energy *E* = 65 eV). Both the 1 × 1 unit cell of the substrate (green, solid line) and the *c*(6 × 2) unit cell of the Ca overlayer are shown. The orange, dashed and red, dotted lines indicate two possible choices for the *c*(6 × 2) unit cell.

The TiO_2_(110) surface was prepared in UHV with sputtering/annealing cycles, as described in detail in the Experimental section, and the surface quality was examined using LEED. In Figure 1, we report the LEED pattern of the TiO_2_ surface: (a) after the usual preparation in UHV resulting in the 1 × 1 clean phase [10] and (b) after calcium segregation due to a higher annealing temperature [1–5].

In the latter case, two sets of diffraction patterns are visible, which is in agreement with the patterns previously observed by other groups for TiO_2_(110) covered with approximately one monolayer (ML) of Ca [1,5]. More intense spots form the rectangular pattern (green, solid line in Figure 1b) characteristic of the clean TiO_2_(110) surface, and correspond to the 1 × 1 termination as shown in Figure 1a. Marked streaks appear along the columns of spots aligned in the 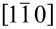 direction, indicating the lack of in-phase correlation as a consequence of some degree of disorder in this direction. This is likely related to oxygen deficiencies in the surface layer caused by UHV preparation [1,10]. Between the primary pattern and the streaks a second set of weaker spots can be observed that form a *c*(6 × 2) superstructure. As proposed by Zhang et al. [1], two possible unit cells,


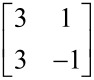


(orange, dashed line) and


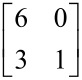


(red, dotted line) are also shown.

**Figure 2:**
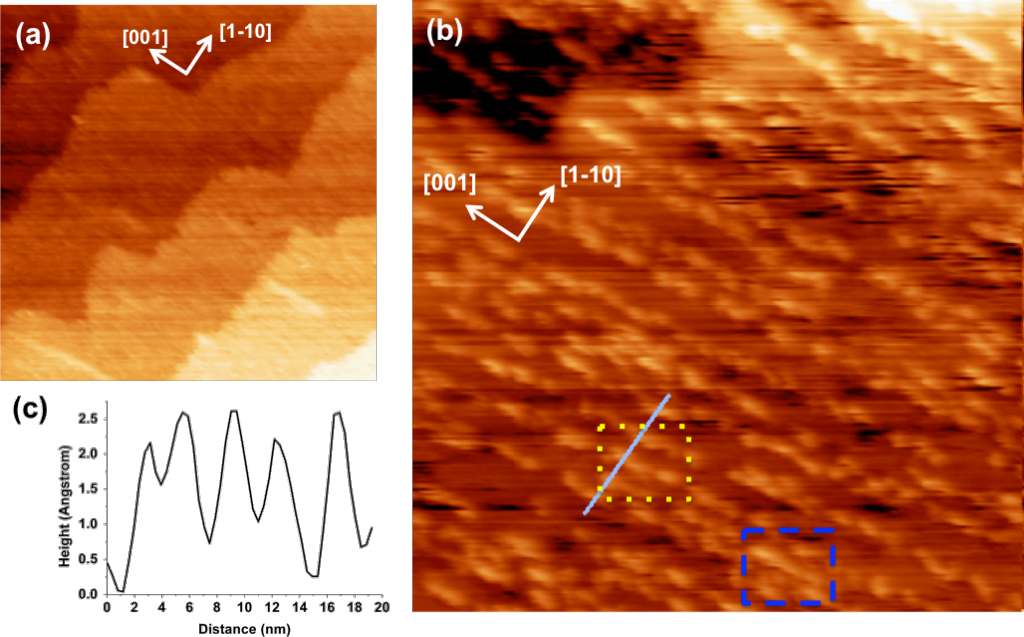
STM images in water of the TiO_2_(110) surface after Ca segregation (a) 370.5 × 370.5 nm^2^, *V*_bias_ = −1 V, *I*_tunnel_ = 1 nA; (b) 81.8 × 81.8 nm^2^, *V*_bias_ = −1 V, *I*_tunnel_ = 1 nA; (c) line profile corresponding to the full line in (b). Ca-related rows of 5× and 3× the spacing (with respect to the TiO_2_(110) periodicity) along the 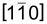 directions are shown in the yellow, dotted and the blue, dashed marked areas, respectively.

The sample was transferred from the UHV chamber to the STM cell and then immersed in high purity water (see Experimental section for details). STM images of the surface shown in Figure 2 were recorded in water after approximately two hours of immersion. In the chosen tip polarity (the sample is grounded), electrons tunnel into empty surface states. The TiO_2_ sample exhibits a terraced surface with a terrace width of about 100 nm, and step height of 3.2 ± 0.5 Å (Figure 2a). This was the expected value for the interplanar distance perpendicular to the (110) surface, and confirms previous STM observations of the clean TiO_2_ surface immersed in bulk water [18]. On the terraces (Figure 2b), rows of ordered bright spots run along the [001] direction of the crystal, their length ranging from a few to tens of nanometers.

Similar rows aligned in the [001] direction were previously observed by STM after Ca deposition or bulk segregation in UHV [1,5]. These are typically up to tens of nanometers long and spaced about three times the periodicity of the substrate in the perpendicular 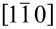 direction. Depending on the surface coverage, these Ca-related, linear features also show different spacings of two, four, or five (and even higher) times the substrate periodicity [1,5]. For a single ML coverage, a *c*(6 × 2) honeycomb lattice was observed on the rutile (110) surface [5]. Additional structures, perpendicular to the primary rows (namely, along the 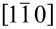 direction) and forming a bi-dimensional network, have been also imaged in UHV on the Ca/TiO_2_ surface below one ML [1,5].

We conclude that the rows imaged in water by STM are consistent with the Ca-related structures due to impurity segregation seen in UHV (see discussion below). In water the mean distance is 3.3 ± 0.2 nm, corresponding to about five times the TiO_2_(110) periodicity along the 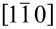 direction (*a*_[1−10]_ = 0.65 nm). Distances equal to three or four, and up to about eight times the substrate periodicity along the 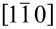 direction were also observed, in agreement with previous UHV results [1,5]. The colored boxes in Figure 2b identify areas where 5× (yellow dotted) and 3× (blue dashed) -spaced rows are present. The resulting irregularity in spacing between Ca-related rows explains the lack of order along the 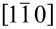 direction in the LEED pattern. From the STM profile of five adjacent Ca structures (Figure 2c, along the full line of Figure 2b) approximately 3 nm apart, a Full Width Half Maximum (FWHM) of 1.2 ± 0.2 nm, and an apparent height of 2.0 ± 0.5 Å were measured, again, in good agreement with previous UHV studies [1,5].

In contrast to the STM studies in UHV, the elongated features along the 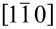 direction were not observed, connecting the rows in a quasi-2D network [1,5]. This could be the consequence of a different quantity of bulk impurities segregated onto the surface in this experiment. This quantity is difficult to control as it depends on the heating temperature and the impurity concentration in the bulk (the latter is unknown in our case). Nevertheless, Bikondoa et al. [5] have reported that in UHV experiments, row-like structures are already visible in the low coverage regime (0.3 ML) along both the [001] and 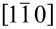 crystallographic directions, below the coverage value we estimate in our images (about 0.5 ML). Thus, we propose that the quasi-one-dimensional appearance of the rows in our data is most likely related to the immersion in water. Dissolution of segregated atoms in liquid very likely depends upon the surface site. Therefore, we hypothesize that the Ca row-like structures along the 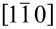 direction [5] are dissolved and transferred into solution before the same process happens for Ca in rows along the [001] direction. We will comment on this assumption below.

**Figure 3:**
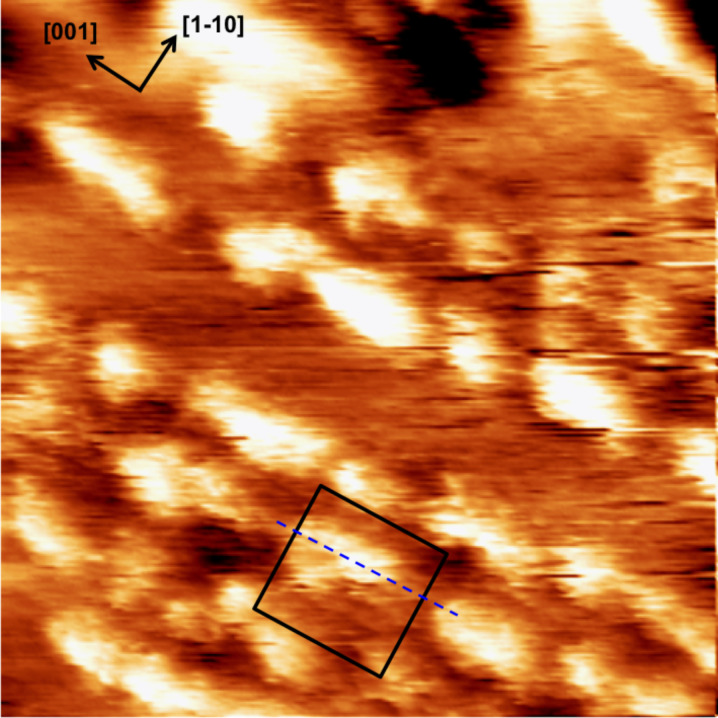
STM image in water of the Ca/TiO_2_(110) surface after Ca segregation, 27.3 × 27.3 nm^2^, *V*_bias_ = −1 V, *I*_tunnel_ = 1 nA. The alignment of the Ca-related rows with TiO_2_(110) substrate rows (dashed, blue line) is highlighted by the black square.

At higher resolution, the rows appear to consist of discrete, elongated or circular, protrusions aligned in the [001] direction (Figure 3), with a length ranging between 1 and 5 nm, and a minimum width of about 1 nm. The smallest protrusions well-exceed atomic dimensions, therefore, a single nanostructure, constituting the row building block, very likely corresponds to a cluster of segregated impurity atoms.

The surface is not completely covered by the nanostructured overlayer: substrate areas free of calcium are visible. We have reported that in atomically resolved images of the rutile (110) surface in water, a 2× periodicity is measured along the [001] direction, suggesting the presence of an adsorbed overlayer (very likely water molecules) [18]. In the present experiment, the quality of the images does not allow for observation of these details. Nevertheless, in the areas free of calcium, weak rows are visible on the substrate and their spacing matches the expected value for the clean TiO_2_(110) 1 × 1 surface (7.0 ± 0.5 Å) [10,18]. In some areas (see for instance the highlighted black square in Figure 3), the Ca-related structures appear to be centered with these substrate rows, as in the UHV experiments [1,5].

**Figure 4:**
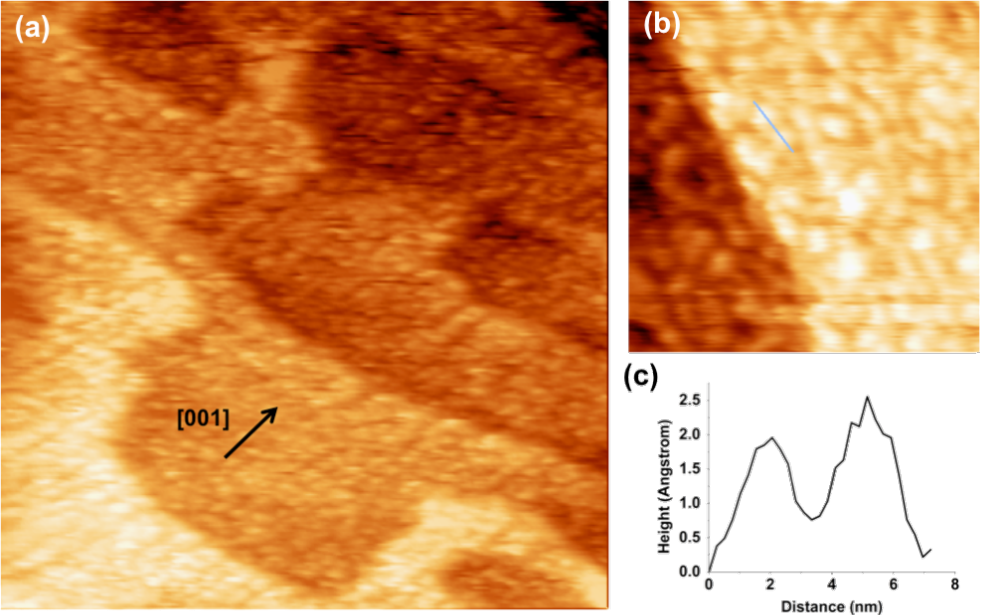
STM images in water of the Ca/TiO_2_(110) surface recorded after a 48 h immersion in the liquid (a) 146.9 × 146.9 nm^2^, *V*_bias_ = −1 V, *I*_tunnel_ = 1 nA; (b) 40.3 × 40.3 nm^2^, *V*_bias_ = −1 V, *I*_tunnel_ = 1 nA; (c) line profile corresponding to the blue line in (b).

The STM images in Figure 2 and Figure 3 were recorded within a few hours of immersion in the liquid. After longer immersion in the aqueous environment, a clear degradation effect of the Ca-related rows in the [001] direction was observed. In Figure 4 we report the resulting images after more than 48 h of continuous immersion in high purity water: the ordered and aligned structures are no longer present on the surface. Although a weak directionality is still discernible in some areas of the surface (see Figure 4a), most of the rounded structures (whose diameter is in the 1–3 nm range) also visible in Figure 3 are now randomly distributed (Figure 4b). A line profile measured across two adjacent protrusions (solid line in Figure 4b) is presented in Figure 4c and shows height and FWHM values very similar to those of the structures assigned to the Ca clusters previously discussed.

XPS analysis carried out after re-entry of the sample in the UHV chamber confirms that calcium is still present on the TiO_2_ surface. Although we did not determine the quantity originally present before immersion in water, this result supports that calcium had segregated to the surface after thermal annealing in UHV, thus enforcing the interpretation of the rows as being Ca-induced.

At present, it is not known which surface sites Ca is preferentially bound to on the rutile TiO_2_(110) surface. Recently, density functional theory (DFT) calculations have shown that, among the possible sites originally proposed on the basis of STM investigations [1], bridging oxygen (BO) atoms and in-plane oxygen (IO) atoms represent minima in the potential energy surface, thus providing more stable sites for adsorption [6]. Interestingly, San Miguel et al. [6] report that from increasing the surface coverage of the surface as well as reducing the proximity to an oxygen vacancy, a significant reduction of the adsorption energy for Ca results. This means that calcium dissolution from the TiO_2_ surface into water should be favored at these sites. Therefore, Ca segregated near a defect site or in the branches directed along the 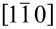 directions (the latter occurring with increasing coverage [6]) disappears first, at the early stages of immersion in water (in our experiment, within 2 h). We note that the average density of surface defects (about 10% [10]) is sufficient to provide the adsorption sites necessary to produce the 2D network observed in UHV [1,5]. The rows which are still on the surface after the first stage of immersion in water are thus formed by Ca bound to the preferred adsorption sites, BO and IO, possibly involving both the Ti and O atoms of the surface layers, and producing a certain degree of local structural deformation [6].

## Conclusion

The Ca-modified TiO_2_(110) rutile surface was investigated in UHV conditions after preparation using sputtering and annealing cycles to produce calcium segregation. The final LEED surface pattern was in agreement with previous studies of calcium overlayers on TiO_2_(110). After transfer from UHV to the liquid cell and immersion in high purity water, STM images in water show a terraced surface with monoatomic steps, in addition to rows aligned with the [001] direction, as expected for Ca segregation in UHV. In contrast to previous UHV results, additional elongated structures in the perpendicular 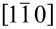 direction are not present, possibly a consequence of a water-induced modification. More than 48 h of continuous exposure to high purity water resulted in a disordered surface with rounded protrusions irregularly distributed over the whole investigated area. The presence of calcium on the surface was confirmed by XPS after reentry of the sample into the UHV chamber.

## Experimental

The TiO_2_ rutile (110) single crystal was purchased from Pi-Kem LTD. The sample was made conductive via bulk reduction in a UHV chamber (P < 1 × 10^−10^ mbar) by means of repeated Ar^+^ sputtering (1 keV) and annealing cycles. This preparation procedure is known to result in the 1 × 1 clean surface [10]. Prolonged sputtering cycles and a higher sample temperature (in our case 1000 ± 50 K) during annealing can cause surface segregation of calcium from the bulk [1–5]. The temperature was monitored by a thermocouple and an infrared pyrometer. The surface structure was examined by LEED. The UHV chamber is equipped with an XPS system. A Mg source and a pass energy of 50 eV were used for the measurements. Peak positions were calibrated according to the O1s position.

After UHV preparation, the sample was taken out of the vacuum chamber and transferred into the STM cell under ultrapure Argon flux. The STM cell was then filled with high purity water (milli-Q purification system, resistivity = 18.2 MΩ∙cm). In order to avoid contamination by oxygen and other gaseous molecules in solution, the milli-Q water was degassed prior to the Argon flux for 1 h. STM measurements were performed using an electrochemical STM [19] equipped with an electrochemical cell positioned in a compact “BEETLE”-type setup [20]. The STM housing was filled with Argon gas in order to ensure an inert atmosphere. Tungsten tips were used for the STM measurements. The tips were prepared by chemical etching (2 M KOH solution) and then coated with hot glue to minimize the faradaic current contribution in the case of electrochemical measurements. The STM images were analyzed with the WSxM software [21].
